# Молекулярные маркеры в генетическом анализе скрещиваемости мягкой пшеницы с рожью

**DOI:** 10.18699/VJ20.649

**Published:** 2020-10

**Authors:** I.V. Porotnikov, O.Yu. Antonova, O.P. Mitrofanova

**Affiliations:** Federal Research Center the N.I. Vavilov All-Russian Institute of Plant Genetic Resources (VIR), St. Petersburg, Russia; Federal Research Center the N.I. Vavilov All-Russian Institute of Plant Genetic Resources (VIR), St. Petersburg, Russia; Federal Research Center the N.I. Vavilov All-Russian Institute of Plant Genetic Resources (VIR), St. Petersburg, Russia

**Keywords:** Triticum aestivum, rye, Kr-genes, QTLs, molecular mapping, molecular-genetic maps, wheat genetic resources, Triticum aestivum, рожь, Kr-гены, QTLs, молекулярное картирование, молекулярно-генетические карты, генетические ресурсы пшеницы

## Abstract

Мягкая пшеница (Triticum aestivum L.), сорта которой широко используются в мировом про- изводстве зерна, плохо скрещивается с видами других родов Triticeae Dum., что ограничивает возмож- ности введения чужеродного генетического материала в ее генофонд и создания новых сортов, хорошо адаптированных к различным неблагоприятным абиотическим и биотическим факторам внешней среды. Известно, что скрещиваемость мягкой пшеницы с представителями других родов контролируется генами Kr1–Kr4 (Crossability with Rye, Hordeum and Aegilops spp.) и геном SKr (Suppressor of crossability). Из названных генов наиболее сильное влияние на признак оказывают SKr и Kr1. В рецессивном состоянии, когда гены не функционируют, может завязываться более 50 % зерновок от числа цветков в колосе при опылении пыль- цой чужеродного вида. Оба гена локализованы в хромосоме 5B. Расположение гена SKr в коротком плече хромосомы 5B ограничено маркерами GBR0233 и Xgwm234 в тесном сцеплении с маркерами Xcfb341, TGlc2 и gene12. Ген Kr1 расположен в длинном плече хромосомы 5B, проксимальнее гена Ph1, между EST-SSR- маркерами Xw5145 и Xw9340. Маркеры, разработанные для гена SKr, применяли для контроля переноса его рецессивного аллеля skr в другие генотипы мягкой пшеницы, что позволило получать формы с высокой за- вязываемостью гибридных зерновок при скрещивании с рожью. Однако в целом использование маркеров генов SKr и Kr1 в практической маркер-ориентированной селекции и молекулярном скрининге образцов ex situ коллекций изучено недостаточно. Большие перспективы в этом плане открывает определение пол- ной нуклеотидной последовательности гена Kr1 у контрастных по скрещиваемости сортов мягкой пшени- цы, это дает возможность создания внутригенных аллель-специфичных маркеров. В представленном обзо- ре рассмотрены генетические ресурсы, созданные посредством гибридизации мягкой пшеницы с рожью; вопросы географического распространения легко скрещивающихся форм пшеницы и генетического кон- троля совместимости пшеницы и ржи; достижения в использовании молекулярных маркеров в картирова- нии Kr-генов и контроле их передачи.

## Abstract (en)

Bread wheat (Triticum aestivum L.), the varieties of which are widely used for the grain production, is difficultly crossable with related species of Triticeae Dum. This factor limits the chance of introduction of alien genetic material into the wheat gene pool and the possibility of new varieties breeding with good adaptation to adverse environmental factors. The crossability between wheat and related species is controlled by Kr1-Kr4 genes (Crossability with Rye, Hordeum and Aegilops spp.) and the SKr gene (Suppressor of crossability). SKr and Kr1 have the largest influence on the trait. In the case of the recessive alleles, these genes do not function and the quantity of hybrid seeds after pollination with alien species can achieve more than 50 %. SKr is located on 5BS between the GBR0233 and Xgwm234 markers, closely linked with the markers Xcfb341, TGlc2 and gene12. Kr1 was mapped on 5BL, proximally to the Ph1 gene, between the EST-SSR markers Xw5145 and Xw9340. The markers of SKr were used to control the transfer of its recessive allele into other wheat genotypes, which made it possible to obtain highly crossable forms. However, the advantages of using the SKr and Kr1 markers in marker-assisted selection and in the screening of ex situ collections are not sufficiently studied. The published Kr1 sequence for varieties with different crossability offers great prospects, because it will be possible to create allele-specific markers. In this review, the following issues are considered: genetic resources created by wheat and rye hybridization, the geographical distribution of easy-to-cross forms of wheat, genetic control of the wheat and rye compatibility, advances of the use of molecular markers in the mapping of Kr-genes and their transmission control.

## Введение

Пшеница – одна из важнейших сельскохозяйственных культур. В 2017 г. в мире было собрано 771.3 млн тонн ее зерна. Россия среди стран-производителей занимает третье место – 85.3 млн тонн (www.fao.org). Успехи в сборе зерна мягкой пшеницы Triticum aestivum L. (2n = 6x = 42, BBAADD) и твердой пшеницы T. durum Desf. (2n = 4x = = 28, BBAA) – это результат интенсивной селекции, на- правленной в основном на повышение урожайности и улучшение качества зерна. Для защиты новых сортов от резких перепадов погоды, происходящих изменений в кли- мате, действия абиотических и биотических стрессоров необходимо иметь соответствующий запас генетической изменчивости у этих культур. Однако в генофондах со- временных селекционных сортов такого запаса изменчи- вости нет. К тому же с середины прошлого столетия по всему миру имеет место процесс обеднения генетического разнообразия селекционных сортов, порожденный ходом исторического развития программ селекции и методами отбора (Porceddu et al., 1988; Kahiluotoa et al., 2019). О на- личии этого процесса свидетельствуют результаты анализа геномов сортов с применением SSR-маркеров во Франции (Roussel et al., 2004), Китае (Hao et al., 2006), Канаде (Fu, Somers, 2009). Чтобы защитить возделываемые пшени- цы от действия различных неблагоприятных факторов, необходимо обогатить их генофонды аллелями генов, увеличивающими разнообразие адаптивных реакций. В качестве источников новых аллелей генов для пшеницы довольно широко используют другие виды родов трибы Triticeae Dum.

Одна из проблем, препятствующих успешным скрещи- ваниям пшеницы с представителями родов Aegilops L., Hordeum L. и Secale L., – наличие прогамной несовме- стимости (Pershina, Trubacheeva, 2017), выражающейся в замедлении или ингибировании роста пыльцевых трубок (Zeven, Van Heemert, 1970; Lange, Wojciechowska, 1976, 1977; Jalani, Moss, 1981). У мягкой пшеницы обнаружено пять генов – Kr1, Kr2, Kr3, Kr4 (Crossability with Rye and Hordeum and Aegilops spp.) (McIntosh et al., 2014) и SKr (Suppressor of crossability) (Tixier et al., 1998), доминант- ные аллели которых отвечают за проявление признака не- совместимости. Эти гены контролируют скрещиваемость пшеницы с видами всех названных выше родов (Snape et al., 1979; Falk, Kasha, 1981; Fedak, Jui, 1982; Sitch et al., 1985; Koba, Shimada, 1993).

Цель настоящего обзора – систематизировать сведения о генетических ресурсах, созданных с помощью межродовой гибридизации мягкой пшеницы с рожью (Secale L.), показать успехи в познании генетического контроля при- знака «легкая скрещиваемость», рассмотреть ДНК-маркеры, которые можно было бы использовать для быстрой и точной идентификации аллелей генов, ответственных за этот признак, и в контроле передачи их в другие генотипы.

## Генетические ресурсы, созданные при скрещивании мягкой пшеницы с рожью, и их использование в селекции

Рожь посевную Secale cereale L. (2n = 2x = 14, геном R) вовлекали в скрещивания с мягкой пшеницей с конца XIX–начала XX веков (Backhouse, 1916). В 1930-х годах в Германии были получены первые формы мягкой пшеницы с транслокациями короткого плеча хромосомы 1R ржи в длинное плечо хромосомы 1B пшеницы (T1BL.1RS). В настоящее время в мире известно более 1050 сортов пшеницы, содержащих эту транслокацию, а также около 100 сортов с транслокацией T1AL.1RS (Rabinovich, 1998; Pershina, 2014; Schlegel, 2019). Широкое распростране- ние обеих транслокаций обусловлено наличием в 1RS комплекса генов, контролирующих устойчивость к раз- личным грибным патогенам, таким как стеблевая (Sr31), бурая (Lr26) и желтая (Yr9) ржавчины, мучнистая роса (Pm8) (Mago et al., 2005; Ren et al., 2009; Crespo-Herrera et al., 2017). Следует отметить, что большая часть сортов с транслокацией T1BL.1RS несет чужеродный хроматин от сорта ржи Petkus из Германии, а с T1AL.1RS и T1DL.1RS – от Insave и Imperial соответственно (Rabinovich, 1998; Schlegel, 2019).

Ограниченное разнообразие ржаного генетического материала, очевидно, не в состоянии обеспечить устойчивость пшеницы к новым расам вредных организмов, которые стали появляться уже в 90-е годы XX в. (Porter et al., 1991; Pretorius et al., 2000). Для решения этой проблемы начали создавать линии пшеницы с новыми ржаными транслокациями путем скрещивания как с теми же сортами ржи, Petkus и Insave, (Ren et al., 2009; Tang et al., 2009), так и с новыми (Ren et al., 2012, 2017; Yang et al., 2014; Li et al., 2016). Кроме того, линию пшеницы, хорошо скрещивающуюся с рожью и несущую T1BL.1RS, ис- пользовали в гибридизации с Secale cereanum cv. Kriszta, и за счет рекомбинации между хромосомами T1BL.1RS и 1R в пшеницу были введены новые аллели генов ржи (Molnár-Láng et al., 2010). Одна из таких рекомбинантных форм дала начало линии M9kr1-Kriszta T1BL.1RS line 179, обладающей устойчивостью к желтой и бурой ржавчинам в сочетании с высоким качеством зерна (Szakács et al., 2020). При использовании совместимых с рожью местных китайских сортов пшеницы Mianyang 11 и A42912 были также созданы линии с T1BL.1RS от разных сортов ржи, которые содержали новые эффективные аллели генов устойчивости к расам Puccinia striiformis f. sp. tritici и Blumeria graminis f. sp. tritici (Ren et al., 2012, 2017; Yang et al., 2014; Li et al., 2016). Передача генетического материала хромосомы 2R ржи в пшеницу сопровождалась переносом генов устойчивости к бурой (Lr25 и Lr45) и стеблевой (Sr59) ржавчинам, повышением адаптивности растений к засушливым условиям климата (Ehdaie et al., 2003). В хромосомах 5RL и 7R обнаружены гены, повышающие эффективность усвоения меди и цинка из почвы (Owuoche et al., 1996; Cakmak et al., 1997). Наиболее полный пере- чень всех известных пшенично-ржаных транслокаций и генов устойчивости приведен в обзоре (Crespo-Herrera et al., 2017). Следует отметить, что в настоящее время потенциал генетического разнообразия, заключенный в хромосомах ржи, помимо 1R, в селекции пшеницы практически остался неиспользованным (Schlegel, 2019).

Считаем нужным подчеркнуть, что в разные периоды времени на базе коллекции Всероссийского института генетических ресурсов растений им. Н.И. Вавилова (ВИР) также проводили исследования по скрещиваемости мягкой пшеницы с рожью. Признак «легкая скрещиваемость пшеницы с рожью» был целенаправленно передан в ози-мый сорт Приекульская 481, на его основе созданы лег- ко скрещивающиеся линии (Суриков, Киссель, 1980), которые наряду с другими линиями использовали в гибридизации с дву- и шестирядными формами ячменя для получения различных межродовых гибридов (Суриков, Киссель, 1987). Созданы оригинальные, хорошо совме- стимые с разными видами ржи формы озимой мягкой пшеницы АМ 808 (с привлечением к-48253 Arthur, США) и МА 808 (к-43920 Мироновская 808, УССР), которые при опылении рожью давали жизнеспособные гибридные и частично фертильные растения F1 (Рехметулин, 1988). Возможность получения частично фертильных гибрид- ных растений и от них константных первичных высоко- продуктивных зимостойких пшенично-ржаных линий подтверждена и в наши дни, при этом методом геномной in situ гибридизации у всех линий обнаружены элимина- ция хромосом генома D и наличие полных геномов B, A и R (Pyukkenen et al., 2019).

## Факторы, влияющие на уровень скрещиваемости пшеницы с рожью

Большая работа по обобщению имеющихся в литературе сведений и результатов собственных исследований по скрещиваемости сортов/линий преимущественно мягкой пшеницы с рожью выполнена A.C. Zeven (1987). На основании собранных нами данных (см. таблицу) можно заключить, что мягкая пшеница в целом плохо скрещи- вается с рожью. Легко (хорошо) скрещивающиеся формы находили с частотой 5.9 %, причем в определенных гео- графических регионах (Lein, 1943; Писарев, 1960; Ригин, 1976; Lange, Wojciechowska, 1976; Falk, Kasha, 1981; Zeven, 1987). Так, выполненная в ВИР оценка сортов мягкой пшеницы различных агроэкологических групп показала, что наиболее высокую способность завязывать гибридные зерновки (52.5 и 53.7 % соответственно) имели сорта ки- тайского и восточносибирского экотипов (Писарев, 1960; Ригин, 1976). Считают, что частое выявление хорошо скрещивающихся форм среди восточноазиатских сортов пшеницы, возможно, связано с тем, что в процессе эво- люции у них не сформировалась генетическая система, препятствующая скрещиваемости пшеницы с рожью, поскольку ареал ржи был ограничен исключительно районами Европы и Западной Азии, и с этой культурой пшеница в Юго-Восточной Азии вместе не произрастала (Lein, 1943; Riley, Chapman, 1967).

**Table 1.**
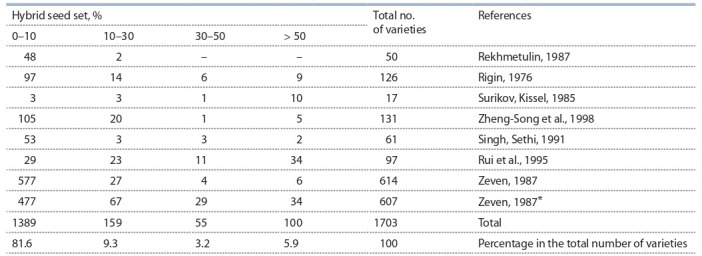
The distribution of wheat varieties by the effectiveness of hybrid seed formation in pollination with rye Note. The intervals for the distribution of varieties into groups are taken from the classical work of A. Lein (1943). * Varieties from other sources cited by A.C. Zeven (1987).

Показано, что на успех скрещиваемости пшеницы с рожью также влияет целый ряд факторов, в том числе видовая принадлежность отцовских и материнских генотипов. Наиболее высокий процент завязываемости ги- бридных зерновок наблюдали у гексаплоидных пшениц: T. aestivum, T. spelta L., T. compactum Host; средний – у те- траплоидных T. turgidum L., T. durum, и самый низкий – у диплоидных T. monococcum L. и T. boeoticum Boiss. Варьировала также успешность применения разных видов ржи в качестве опылителей: высокая скрещиваемость при опылении пыльцой диплоидных видов S. cereale и S. vavilovii Grossh., низкая – тетраплоидного вида S. fragile M. Bieb. (Ригин, 1965, 1976; Суриков, Киссель, 1985; Рехметулин, 1987; Molnár-Láng, 2015). Различные аспекты проблемы скрещиваемости пшеницы с рожью и други- ми видами рассмотрены в обзорной статье (Molnár-Láng, 2015).

## Гибридологический анализ скрещиваемости пшеницы с рожью и локализация генов легкой скрещиваемости

Первые сведения о наследовании признака «легкая скре- щиваемость мягкой пшеницы с рожью» получены немец- ким ученым A. Lein (1943). Выяснение характера насле- дования признака у реципрокных гибридов F_1_–F_3_ между сортом Chinese 466 из Аргентины, характеризующимся высокой (57.3 %) завязываемостью гибридных зерновок, и плохо скрещивающимися с рожью сортами Marguis (Ка- нада), Peragis и Blausamtiger Kolben (Германия) с завязы- ваемостью 1.9, 2.5 и 12.7 % соответственно, показало в F1 доминирование низкой завязываемости, а в F3 – дигибрид- ное расщепление. Аллельные пары факторов (генов) были обозначены как Kr1-kr1 (Kreuzbarkeitsgene) и Kr2-kr2, а генотипы родительских сортов – kr1kr1kr2kr2 (Chinese 466), Kr1Kr1Kr2Kr2 (Marquis и Peragis) и Kr1Kr1kr2kr2 (Blausamtiger Kolben). Отмечено также, что доминантный аллель Kr1 обладает более сильным ингибирующим эффектом роста пыльцевых трубок, чем Kr2, а различные генотипы характеризуются следующим процентом за- вязываемости гибридных зерновок: Kr1Kr1Kr2Kr2 – до 10 %, Kr1Kr1kr2kr2 – 10–30, kr1kr1Kr2Kr2 – 30–50, kr1kr1kr2kr2 – более 50 % (Lein, 1943).

Для установления хромосомной локализации генов Kr1 и Kr2 была использована серия из 21 линии с меж- сортовым замещением хромосом Chinese Spring/Hope (Sears et al., 1957; Riley, Chapman, 1967). При опылении диплоидной рожью у Chinese Spring (далее CS) наблюдали высокую (74.3 %) завязываемость гибридных зерновок, а сорт Hope таких зерновок практически не образовал. Существенное снижение уровня завязываемости зерновок обнаружено у линий с замещенными хромосомами 5А и 5В, хотя различия между линиями не имели статистиче- ского подтверждения (см. рисунок). Полученные данные навели R. Riley и V. Chapman на мысль, что замена хро- мосом 5B вызывает бóльшую редукцию уровня скрещи- ваемости, чем хромосом 5А. Ген Kr1 был локализован в хромосоме 5В, а Kr2 – в 5А (Riley, Chapman, 1967). Чтобы ответить на вопрос, рецессивные аллели Kr-генов поддерживают межродовую совместимость или, напро- тив, доминантные аллели ингибируют скрещиваемость, авторы процитированной работы протестировали линию CS nulli-5B-tetra-5D, у которой отсутствовали хромосомы 5В, но присутствовала в учетверенной дозе гомеологич- ная ей хромосома 5D. Эта линия, как и моносомик CS по хромосоме 5B, формировала зерновки при самоопыле- нии. При опылении рожью в первом случае 116 цветков дали 67 гибридных зерновок (завязываемость 57.8 %), во втором – при опылении 261 цветка было получено 184 зерновки (70.5 %). Отсутствие хромосом 5B и, соот- ветственно, пары рецессивных аллелей kr1 или изменение их дозы не снижало уровень завязываемости гибридных зерновок. Из этого R. Riley и V. Chapman сделали вывод, что рецессивные аллели не активны, доминантные аллели гена Kr1 действуют как ингибиторы скрещиваемости. По- скольку линии CS/Hope 5A и CS/Hope 5B, в отличие от сорта Hope, не теряли полностью способности образовы- вать гибридные зерновки при опылении рожью, эффекты ингибиторов Kr1 и Kr2, по мнению этих ученых, были или аддитивными, или комплементарными.

**Fig. 1.**
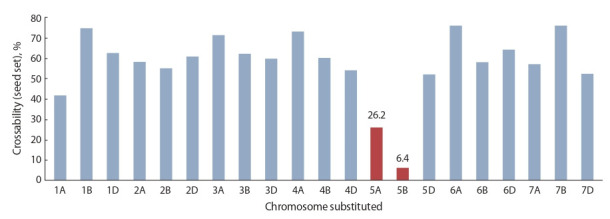
Crossability of wheat intervarietal substitution lines of Chinese Spring/Hope to rye (according to Riley, Chapman, 1967).

Нужно отметить, что о возможном кумулятивном действии генов Kr1 и Kr2 ранее сообщил A. Lein (1943): гетерозиготные генотипы Kr1kr1kr2kr2 и kr1kr1Kr2kr2 имели более 10 % успешных скрещиваний с рожью, тогда как часть растений с генотипами Kr1Kr1Kr2kr2 и Kr1kr1Kr2Kr2 не завязала ни одной гибридной зерновки. Впоследствии это предположение подтвердили результаты работы (Falk, Kasha, 1983), было отмечено также, что рецессивные аллели kr1 и kr2 кумулятивным эффектом не обладали.

Для картирования гена Kr1 в хромосоме 5В использо- вали гибриды, полученные от скрещивания замещенной линии CS/Hope 5B, имеющей низкую завязываемость зерновок при опылении рожью, с дителосомной линией CS DT5BL, у которой хромосома 5B была представлена парой телоцентриков по ее длинному плечу, а короткое плечо отсутствовало (Lange, Riley, 1973). По способности завязывать гибридные зерновки эта линия не отлича- лась от сорта CS. Средняя частота рекомбинации между Kr1 и центромерой составила 11.45 ± 3.0 %. Локализацию гена Kr1 в длинном плече хромосомы 5B подтвердили L.A. Sitch с коллегами (1985), однако ген располагался на большем расстоянии от центромеры – 44.8 ± 3.28 % рекомбинации. Причиной такого несоответствия могло быть использование другой родительской формы – сорта Highbury.

Ген Kr2 картировали у гибридов, полученных от скре- щивания CS с линией CS/T. spelta 5A, у которой пара хро- мосом 5A мягкой пшеницы замещена на пару хромосом 5A от T. spelta (Sitch et al., 1985). Ген Kr2 оказался тесно сцепленным (частота рекомбинации 4.8 ± 4.66 %) с геном Vrn1 (Response to Vernalization), определяющим яровой тип развития растений пшеницы, и был расположен дистальнее гена Q (Squarehead/spelt), обусловливающего образование спельтоидного типа колоса (McIntosh et al., 2014), на расстоянии 38.1 ± 10.60 % рекомбинации от него (Sitch et al., 1985). Примерно одинаковое положение Kr1 и Kr2 по отношению к центромере авторы рассматривали как доказательство в пользу гомеоаллельности этих генов. Кроме того, в данной работе статистически значимые различия по завязываемости гибридных зерновок были выявлены у эуплоидных растений сорта CS и у получен- ных на его основе моносомиков mono-5B и дителосомных линий DT5BL. Эуплоидные растения CS завязывали в два раза меньше зерновок, чем растения двух упомянутых линий. Этот эффект L.A. Sitch с коллегами (1985) связали с уменьшением дозы короткого плеча хромосомы 5B и предположили присутствие в нем супрессора скрещивае- мости. Позднее наличие такого супрессора, получившего название SKr (Supressor of crossability), было доказано методами молекулярно-генетического картирования (Tixier et al., 1998).

Еще один ген, Kr3, был картирован у гексаплоидной пшеницы в хромосоме 5D (Krolow, 1970). У доминантного аллеля этого гена эффект ингибирования скрещиваемости был слабее, чем у Kr1 и Kr2. При гибридизации с рожью влияние Kr3 зачастую оказывалось статистически незна- чимым, и в ряде работ (Riley, Chapman, 1967; Falk, Kasha, 1983; Sitch et al., 1985; Zheng et al., 1992) участие хромо- сомы 5D в контроле завязываемости пшенично-ржаных гибридных зерновок не подтверждено. Однако действие Kr3 как ингибитора межродовой совместимости удалось продемонстрировать при гибридизации с Hordeum bulbosum L. на линиях CS/Hope 5D и CS/Hope 5A, получив- ших от сорта-донора Hope хромосомы с доминантными аллелями генов Kr3 и Kr2 соответственно (Snape et al., 1979). Снижение скрещиваемости с H. bulbosum у этих замещенных линий было примерно одинаковым, т. е. эффект гена Kr3 был равен эффекту гена Kr2.

Ген Kr4 обнаружен моносомным анализом линии мяг- кой пшеницы J-11 из провинции Сычуань (Китай). Эта линия показала почти 100 % завязываемость гибридных зерновок при опылении рожью (Zheng et al., 1992). На- следование признака хорошей скрещиваемости изучали у гибридов F1 между линией J-11 и моносомными лини- ями сортов Abbondanza (скрещиваемость с рожью менее 2.13 %, предположительный генотип Kr1Kr1Kr2Kr2) и Chinese Spring (генотип kr1kr1kr2kr2). В первом случае присутствие хромосом 1А, 5А и 5В от линии J-11 ока- зывало положительное влияние на успех скрещивания с рожью, в то время как у гибридов с моносомиками CS для тех же хромосом наблюдали снижение скрещиваемо- сти. При этом влияние хромосомы 1A на завязываемость зерновок оказалось сильнее, чем 5A (kr2), но слабее, чем 5B (kr1). Это позволило Y.L. Zheng с коллегами (1992) сделать вывод о наличии в хромосоме 1А линии J-11 дополнительного гена kr4, сходного по действию с kr1 и kr2. Точная хромосомная локализация kr4 пока остается неизвестной.

## Молекулярно-генетическое картирование гена SKr и поиск локусов, влияющих на уровень скрещиваемости пшеницы с рожью

Новые возможности для точной локализации генов легкой скрещиваемости пшеницы с рожью возникли в связи с введением в генетический анализ технологии молекулярного маркирования и созданием насыщенных молекулярно-генетических карт (Xie et al., 1993; Nelson et al., 1995; Cadalen et al., 1997; Röder et al., 1998; Somers et al., 2004; Leonova, 2013; Khlestkina, 2014). Результаты работ разных исследователей по молекулярным маркерам и картам хромосом мягкой пшеницы и других видов трибы Triticeae доступны в цифровой базе данных GrainGenes (https://wheat.pw.usda.gov/GG3/).

Основополагающая работа по определению локусов количественных признаков (QTLs), влияющих на скре- щиваемость, выполнена с использованием маркеров RFLP на популяции из 187 DH-линий, производных от гибрида CS×Courtot (Tixier et al., 1998). Скрещиваемость родитель- ских форм и всех DH-линий проверяли путем опыления пыльцой ржи сорта Dankowskie Nowe, высокий уровень скрещиваемости был подтвержден для CS (95 %, генотип kr1kr1kr2kr2), низкий – для Courtot (10 %, Kr1Kr1kr2kr2) (Gay, Bernard, 1994). Для локализации в хромосомах QTLs, оценки их аддитивных эффектов и эффектов доминиро- вания M.H. Tixier с коллегами (1998) применяли метод логистической линейной регрессии, который приложим к линиям, полученным от F1 путем самоопыления или бек- кроссирования, и DH-линиям, при условии, что имеется информация об их генотипах по RFLP, RAPD или другим типам маркеров (Kearsey, Hyne, 1994). О местоположении QTLs судили по отношению величины аддитивного эф- фекта к фенотипической дисперсии:

avp = a^2^/σ^2^_p_,

где а – аддитивный эффект, σ^2^_p_ – фенотипическая диспер- сия, p – вероятность успешного скрещивания.

Всего в хромосомах 5B и 7A у DH линий было обнару- жено три локуса от сорта Courtot, подавляющие способ- ность скрещиваться с рожью. При этом в хромосомах 5A (Kr2) и 5D (Kr3) таких локусов не найдено. Главный локус (avp = 16.8 %) находился в дистальном районе короткого плеча хромосомы 5B вблизи маркера Xf ba367, который имел наибольший аддитивный эффект (a = 13.6) и был тесно сцеплен (2.5 сМ) с маркером Xpsr170. Дру-гой локус, ассоциированный с маркером Xtam51 (хро- мосома 7AL), оказывал значительный аддитивный эф- фект (a = 8.1) при рассмотрении его в модели вместе с Xf ba367. Еще один QTL, влияющий на скрещиваемость и, по-видимому, относящийся к гену Kr1, был картирован в длинном плече хромосомы 5В и фланкирован RFLP-маркером Xwg583 (a = 6.1; avp = 3.3 %) и SSR-маркером Xgwm271. Этот QTL оказывал более слабое влияние на фенотипическое проявление признака, для расчета его эффекта понадобилось привлечь методы маркерной логистической регрессии с двумя QTL в хромосоме 5B (Tixier et al., 1998).

Ранее в процедурах по картированию Kr-генов изуча- ли расщепление в потомствах гибридов от скрещивания дителосомной по длинному плечу хромосомы 5B линии CS (kr1kr1) с замещенной линией CS/Hope 5B или сортом Highbury, имеющими доминантные аллели гена Kr1, и рассчитывали частоту рекомбинации между kr1 и центромерой. Влияние короткого плеча хромосомы 5B CS на скрещиваемость не обнаружено (Lange, Riley, 1973; Sitch et al., 1985). Однако в работе L.A. Sitch с коллегами (1985) показано, что уменьшение дозы 5BS сопровождалось повышением уровня скрещиваемости CS с H. bulbosum. Выявление у гибрида CS×Courtot в коротком плече 5B локуса, существенно влияющего на уровень скрещивае- мости с рожью, дало основание предположить наличие у сорта Courtot еще одного доминантного гена, подавляющего межродовую гибридизацию. Ген-супрессор был обозначен как SKr (Supressor of crossability) (Tixier et al., 1998). Работа по его молекулярному картированию вы- полнена D. Lamoureux с коллегами (2002). Анализ QTLs проводили на основе тех же фенотипических данных, что были получены M.H. Tixier с коллегами (1998), но для картирования использовали преимущественно маркеры AFLP. Молекулярная карта хромосомы дистального участ- ка 5BS была увеличена на 16.1 сМ путем интегрирования маркеров E36M49-287 (после секвенирования назван Xdl103) и E32M61-233. Несколько маркеров были также интегрированы в хромосомную карту 5B в район центро- меры. Анализ подтвердил наличие в 5BS главного QTL (a = 15.9 %; avp = 22.1 %), ингибирующего скрещиваемость с рожью, который наиболее вероятно расположен на участке размером в 5.1 сМ между маркерами Xdl103 и Xf ba367 и на расстоянии 12.7 сМ от дистального конца хромосомы. Поиск областей, ортологичных гену SKr, был проведен посредством скрининга библиотеки BAC-клонов риса с помощью зондов Xpsr170 и Xdl103 (клонирован- ный маркер AFLP E36M49-287), картированных вблизи SKr. В хромосомах 5 и 6 генома риса обнаружены QTLs с функциями, близкими к SKr, в том числе: локус f5, от- ветственный за фертильность (Wang et al., 1998), локус S5, контролирующий стерильность у межсортовых гибридов (Liu et al., 1997), а также локус S_10_, детерминирующий мужскую стерильность (Lorieux et al., 2000).

Создание насыщенной генетической карты для района хромосомы 5BS с геном SKr получило продолжение в работе W. Alfares с коллегами (2009). Они изучали по- пуляцию линий-потомков F6 от самоопыления гибридов MP98×Courtot. Родительская линия MP98 была отобрана по признаку высокой скрещиваемости с рожью (70 ± 11.3 %) из популяции дигаплоидов гибрида CS×Courtot (Tixier et al., 1998; Lamoureux et al., 2002). Для картирования были применены новые маркеры из опубликованных баз данных: для хромосомы 5BS было взято восемь маркеров, детектирующих полиморфизм у родительских форм, включая микросателлиты Xgwm234, Xgpw4098, Xwmc149 и Xgwm443, все они располагались прокси- мально по отношению к гену SKr (Alfares et al., 2009). Среди них Xgwm234 был локализован довольно близко к SKr (0.2 сМ). Для насыщения карты и поиска маркеров в дистальной области привлекли дополнительные маркеры с EST-карты генома ячменя (Stein et al., 2007) как наиболее родственного пшенице злака. В результате были найдены два EST-маркера, GBR0233 и GBR1541, картированные как RFLP-зонды дистально и проксимально от SKr соот- ветственно.

Секвенированная последовательность маркера GBR0233 оказалась гомологична участку гена риса Os12g44150 (хромосома 12L) (Alfares et al., 2009), который, в свою очередь, относится к группе генов Os12g44180– Os12g44250, иден- тифицированных в другой работе как гомологи гена GSP (Grain softness protein = белок мягкозерности зерновки) мягкой пшеницы (Chantret et al., 2004). На основе после- довательности BAC клона 1793L02 (GenBank #CT009585), содержащего ген GSP, авторы разработали сцепленные с геном SKr SSR-маркеры Xсf b306 и Xcf b309. Кроме того, последовательность RFLP-маркера GBR0233 проявляла гомологию с двумя EST-контигами мягкой пшеницы, один из которых, CTG_WHP_856.1-G356.103K22R011024, содержал специфичную для хромосомы 5В последова- тельность ATPase1-5B, для ее детекции также была раз- работана пара праймеров (Alfares et al., 2009).

Маркеры Xсf b306, Xcf b309, ATPase1-5B применяли для скрининга библиотеки BAC-клонов сорта CS. Были выделены BAC-клоны, 2163O14 и 317L24А, расположен- ные проксимально и дистально по отношению к области SKr соответственно. Секвенирование этих клонов дало информацию для разработки дополнительных ISBP- и SSR-маркеров. Определены 5B-специфичные SSR-маркеры Xcf b341 и Xcf b382, тесно сцепленные с локусом SKr. Таким образом, ген SKr был картирован в интервале 0.1 сМ с тесно сцепленными SSR-маркерами Xcf b306, Xcf b382, Xcf b341 и фланкирован в промежутке 0.3 сМ между GBR0233 (проксимальное положение) и Xgwm234 (дистальное положение) (Alfares, 2009; Alfares et al., 2009). Позднее была показана достоверная ассоциация гена SKr и маркеров Xcf b341, TGlc2 (ISBP), gene12 и gene13, по- следние два получены на основе ортологичных генов риса Os12g44100-1 и Os12g44090 соответственно (Bouguennec et al., 2018). Маркеры Xcf b306 и Xcf b341 использовали при картировании гена SKr и в других популяциях, например популяции F_2_ (Renan×CS) (Alfares et al., 2009), а также для молекулярно-генетического картирования хромосомы 5B (Timonova et al., 2013).

Другая попытка картирования генов легкой скрещиваемости мягкой пшеницы с рожью предпринята в работе японских исследователей (Mishina et al., 2009). Для карти- рования использовали рекомбинантные по хромосоме 5B инбредные линии (CS×CS/Cheyenne5B) и 81 SSR-маркер из работы D.J. Somers с коллегами (2004) и интернет-ре- сурса Wheat composite 2004 map at GrainGenes 2.0 (https://wheat.pw.usda.gov/GG3/). Включение в анализ описанных ранее маркеров Xcf b341 и Xgwm234 (Tixier et al., 1998; Lamoureux et al., 2002; Alfares et al., 2009) не дало поло- жительных результатов из-за отсутствия полиморфизма у родительских форм. В этой популяции основной по силе QTL (SKr) был тесно сцеплен с SSR-маркером Xgwm443 хромосомы 5BS и ограничен генетическими локусами Xcfd5 и Xbarc216 (интервал 50 сМ), расположенными дистально и проксимально соответственно (Mishina et al., 2009). Список молекулярных маркеров, сцепленных с геном SKr, приведен в Приложении^1^.

^1^ Приложение см. по адресу: http://www.bionet.nsc.ru/vogis/download/pict-2020-24/appx7.pdf



Возможность практического использования маркеров гена SKr в маркер-ориентированной селекции (marker assisted selection, MAS) продемонстрирована в работах (Alfares et al., 2009; Bouguennec et al., 2018). Так, на этапах получения гибридов от скрещивания линии Ct(FK5B), у которой пара хромосом 5B Courtot была замещена на пару гомологов от японского сорта Fukuho Komugi, имеющего хорошую скрещиваемость, с шестью сортами мягкой пшеницы с плохой скрещиваемостью и проведения беккроссов BC_2_F_2_ наличие/отсутствие рецессивных ал- лелей skr контролировали с помощью маркеров Xcf b306 и Xcf b341. Практически все гибридные линии показали полное соответствие между наличием диагностических фрагментов маркеров Xcf b306 и Xcf b341 и ожидаемым уровнем скрещиваемости с рожью (Alfares et al., 2009)

В работе A. Bouguennec с коллегами (2018) для контроля передачи рецессивных аллелей skr от двух линий, полу- ченных W. Alfares с коллегами (2009), в сорт пшеницы Barok использовали как уже известные SSR-маркеры Xcf b306, Xcf b341 и Xgwm234, так и в дополнение к ним новые разработанные маркеры TGlc2 (ISBP), gene12 и gene13. Маркеры TGlc2, Xcf b341 и gene12 косегрегиро- вали с SKr. На каждом этапе скрещиваний (до BC_3_F_2_) гибридные формы тестировали на наличие молекулярных маркеров, благодаря чему подтвердили возможность их использования в контроле передачи рецессивного аллеля skr в другие сорта. Кроме того, диагностическая эффек- тивность маркеров Xcf b341, TGlc2 gene12 и gene13 была проверена при генотипировании 15 сортов и линий мягкой пшеницы различного географического происхожде- ния. По результатам молекулярного скрининга, 11 из них (12 по маркеру gene13) имели правильную ассоциацию «маркер-признак», а для четырех сортов с плохой скре- щиваемостью (трех в случае gene13) соотнести признак и диагностические фрагменты маркеров не удалось – про- дукты амплификации у них по размеру соответствовали аллелю «легкой скрещиваемости» (Bouguennec et al., 2018).

## Молекулярно-генетическое картирование гена Kr1

Сначала местоположение гена Kr1 было определено на расстоянии 20 сМ от гена-супрессора гомеологичного спаривания хромосом Ph1, расположенного в прокси- мальной области хромосомы 5BL (Snape et al., 1995). По результатам последующих исследований (Lamoureux et al., 2002) Kr1 был локализован на расстоянии 90.3 сМ от дистального конца 5BL между маркерами E33M60-233 (AFLP) и Xgwm271 (SSR), при этом наблюдали независи- мое расщепление потомства по генам Kr1 и SKr. Эффект QTL, который был соотнесен с Kr1, оказался намного слабее (a = 7.9 %; avp = 5.5 %) эффекта SKr (a = 15.9 %; avp = 22.1 %).

Для уточнения локализации гена Kr1 I. Bertin с колле- гами (2009) использовали популяцию рекомбинантных линий, полученных от скрещивания сорта Hobbit sib, не- сущего реципрокно-транслоцированные плечи хромосом 5BL-7BL и 5BS-7BS, с замещенной линией Hobbit sib (CS 5BL, 7BL), у которой длинные плечи хромосом 5B и 7B были замещены на гомологи от CS. Показано, что Kr1 рас- положен в области 13 сМ между SSR-маркерами Xgwm213 и Xgwm371. С помощью этих маркеров у мутантной линии ph1b подтверждена локализация вблизи Ph1 гена Kr1. Сведения о локализации Ph1 и установленная синтения содержащего его участка по отношению к хромосомам 9 риса и Bd4 коротконожки Brachypodium distachyon L. позволили привлечь новые маркеры для тонкого картиро- вания Kr1 и выявить два локуса, влияющих на скрещиваемость с рожью. Один из них расположен проксимально к гену Ph1 на расстоянии не более 2 сМ и фланкирован EST-SSR-маркерами Xw5145 (дистальное положение) и Xw9340 (проксимальное положение). Этот район также включает SSR-маркер Xgwm213, ранее примененный для картирования Kr1. Другой участок – Crossability region 2, расположенный дистально от Ph1, оказался более важным для тонкого картирования, поскольку имел выше уровень рекомбинации, а также синтению с участком хромосомы Bd4 у B. distachyon размером 2.5 Mб. Участок Crossability region 2 ограничен в интервале 14 сМ ESTSSR- маркерами 1275L15_cg3 и Os09g38060 и включает SSR-маркер Xgwm371. Линии с рекомбинацией по этому участку могли скрещиваться с рожью даже при наличии доминантных аллелей гена Kr1. Нужно отметить, что пер- воначальное картирование Kr1 на участке в 13 сМ между SSR-маркерами Xgwm213 и Xgwm371 не позволило до- казать присутствие второго локуса. Перечень молекулярных маркеров, сцепленных с локусами Kr1 и Crossability region 2, представлен в Приложении.

В настоящее время удалось определить нуклеотидную последовательность доминантного (от сорта Mazhamai) и рецессивного (от CS) аллелей гена Kr1. Основой для этого послужили работы японских исследователей (Manickavelu et al., 2009a, b), изучивших экспрессию генов в тканях пестиков у рекомбинантных инбредных линий, различающихся по скрещиваемости с рожью, при этом впервые получены дифференциально экспрессированные фрагменты кДНК (differentially expressed fragment, DEF), среди которых, возможно, были фрагменты гена Kr1. Пу- тем сравнения полученных DEF-последовательностей с последовательностями баз данных генбанков DDB Blastx и NCBI Blast обнаружены по меньшей мере две нуклеотидные последовательности, относящиеся к гену Kr1. Фрагмент DEF1 (#AB289691) был идентифицирован как гомолог гена ZmPti1a, который входит в группу PTI1- подобных киназ кукурузы (Pti1-like kinases) и участвует в стимулировании прорастания пыльцевых трубок. По- следовательность DEF2 (#AB379558.1) была гомологична гену кальций-зависимой протеинкиназы риса – CDPK (Calcium-dependent protein kinase) – белку, участвующему в передаче различных стресс-чувствительных сигналов при развитии цветка (Manickavelu et al., 2009a, b).

На основе опубликованной последовательности кДНК гена Kr1 (#AB379558.1) китайские ученые с помощью специфичных праймеров амплифицировали фрагменты этого гена у трех сортов пшеницы (Cai et al., 2012). Полу- ченные последовательности на 85 % были гомологичны гену SRK (S-locus receptor kinase) (Stein et al., 1991), который экспрессируется в тканях пестика и отвечает за распознавание пыльцы у вида Brassica oleracea L. Наряду с другими генами S-локуса, SRK участвует в контроле совместимости рыльца пестика с чужеродной пыльцой.

На базе той же частичной последовательности гена Kr1 (#AB379558.1) были разработаны наборы праймеров, по- зволившие провести полное секвенирование этого гена с помощью методов прогулки по геному (Genome walking) и быстрой амплификации концов кДНК (Rapid amplification of cDNA ends) (Cai et al., 2016). У плохо скрещивающего- ся с рожью сорта Mazhamai (Kr1/–) последовательность гена имеет длину 4006 п. н. и содержит три интрона, че- тыре экзона общей длиной 1671 п. н., которые кодируют 557 аминокислот. У сорта CS (kr1/kr1) последовательность гена короче (3945 п. н.) и только на 56.24 % гомологична доминантной аллели. Основные отличия рецессивного аллеля от функционального отмечены в третьем и чет- вертом экзонах, которые у kr1 содержат большое количе- ство стоп-кодонов, что указывает на невозможность его экспрессии.

## Другие гены скрещиваемости пшеницы с рожью

Все описанные выше гены и QTLs, ответственные за скрещиваемость с рожью, характерны для мягкой пше- ницы. Синтетическая гексаплоидная пшеница имеет тот же геномный состав, что и мягкая, но обладает куда более широким генетическим разнообразием, поскольку для ее получения в качестве родительских форм привлека- ют различных представителей тетраплоидных пшениц (2n = 4x = 28, BBAA) и вида Ae. tauschii (Хакимова и др., 2019). Это позволяет предположить, что у синтетической пшеницы могут присутствовать другие локусы, эффект которых будет схож с генами семейства Kr.

Действительно, такие гены обнаружены у синтетиче- ской гексаплоидной пшеницы Am3, полученной путем гибридизации пшеницы карталинской T. carthlicum Nevski = T. persicum Vav. с Ae. tauschii (Zhang et al., 2011). После скрещивания Am3 с китайским сортом пшени- цы Laizhou953 при последующем многократном бек- кроссировании и самоопылении гибридов популяция из интрогрессивных линий BC5F6 была подвергнута молекулярному скринингу с использованием 1256 SSR- маркеров. Всего было найдено 13 QTL, для 5 из них, QCa. caas.1A, QCa.caas.2D, QCa.caas.4B, QCa.caas.5B и QCa.caas.6A, влияние на скрещиваемость установлено в обеих провинциях. Наиболее сильный локус QCa. caas.5B в хромосоме 5BS был сцеплен с SSR-маркером Xwmc149 (унаследован от T. carthlicum), что позволило ассоции- ровать его с геном SKr. Дополнительные локусы отмечены в хромосомах 1A, 2D, 4B и 6A. Локус QCa.caas.1A, полученный также от пшеницы карталинской и флан- кированный SSR-маркерами Xbarc213 (проксимальный) и Xbarc287 (дистальный) в интервале 10 сМ в коротком плече хромосомы 1A, интересен тем, что он может от- носиться к Kr4. Как утверждают L. Zhang с коллегами (2011), три других локуса – QCa.caas.2D (унаследован от Ae. tauschii), QCa. caas.4B и QCa.caas.6A (унаследованы от Laizhou953) – были открыты впервые.

## Заключение

Насущная необходимость обогащения генофонда пше- ницы новыми аллелями генов, прежде всего контроли- рующими различные адаптивные реакции растений, стимулирует проведение исследований по поиску новых форм, легко скрещивающихся с представителями родов Secale, Aegilops и Hordeum, а также по изучению генетики межродовой совместимости. Полученные новые знания о генетической природе признака «легкая скрещивае- мость» напрямую связаны с введением в генетический анализ разных типов молекулярных маркеров, построени- ем насыщенных генетических карт хромосом пшеницы, молекулярно-генетическим картированием Kr-генов и идентификацией QTLs, влияющих на успех межродовой гибридизации, созданием доступного для изучения ис- ходного материала и формированием общего информа- ционного банка данных.

В настоящее время на молекулярно-генетических картах локализованы гены SKr и Kr1 и разработано значи- тельное число ассоциированных с ними маркеров. Однако примеры применения этих маркеров в маркер-ориентиро- ванной селекции пока немногочисленны. Как сообщается в литературе, с определенной долей успеха эффективны тесно сцепленные и косегрегирующие с геном SKr марке- ры TGlc2, Xcf b341 и gene12. В качестве дополнительных маркеров могут выступать gene13, Xcf b306 и Xgwm234. С их помощью удалось отследить интрогрессию участка хромосомы, ассоциированного с легкой скрещиваемостью, в отдельные сорта пшеницы, что во многом ускорило процесс создания желаемых форм. Эффективность маркеров, сцепленных с геном Kr1 и районом Crossability region 2, в контроле такой передачи, а также для скрининга ex situ коллекций пшеницы только предстоит проверить. Большие перспективы открываются в связи с опублико- ванной недавно полной нуклеотидной последовательно- стью гена Kr1, что дает возможность разработки внутри- генных аллель-специфичных маркеров.

Поиск генов легкой скрещиваемости с рожью про- должается. Примером может послужить синтетическая гексаплоидная пшеница Am3, где с помощью молекуляр- ных маркеров обнаружено три новых QTL. Созданные путем гибридизации пшеницы с рожью разного типа генетические линии, сорта, содержащие транслокации и интрогрессии с чужеродными аллелями генов, формируют ценный ресурс для проведения научных исследований и селекции.

## Conflict of interest

The authors declare no conflict of interest.
